# Cascade and Fusion of Multitask Convolutional Neural Networks for Detection of Thyroid Nodules in Contrast-Enhanced CT

**DOI:** 10.1155/2019/7401235

**Published:** 2019-10-20

**Authors:** Zuopeng Zhao, Chen Ye, Yanjun Hu, Ceng Li, Xiaofeng Li

**Affiliations:** ^1^School of Computer Science and Technology & Mine Digitization Engineering Research Center of Ministry of Education of the People's Republic of China, China University of Mining and Technology, Xuzhou 221116, China; ^2^School of Information and Control Engineering, China University of Mining and Technology, Xuzhou 221116, China; ^3^Department of Computed Tomography, Xuzhou Third People's Hospital, Xuzhou 221116, China

## Abstract

With the development of computed tomography (CT), the contrast-enhanced CT scan is widely used in the diagnosis of thyroid nodules. However, due to the artifacts and high complexity of thyroid CT images, traditional machine learning has difficulty in detecting thyroid nodules in contrast-enhanced CT. A fully automated detection algorithm for thyroid nodules using contrast-enhanced CT images is developed. A modified U-Net architecture of fully convolutional networks is employed to segment the thyroid region of interest (ROI), and a fusion of convolutional neural networks (CNN-Fs) is proposed to detect benign and malignant thyroid nodules from the ROI images and original contrast-enhanced CT images. Experimental results demonstrate that the proposed cascade and fusion method of multitask convolutional neural networks (CNNs) is efficient in diagnosing thyroid diseases with contrast-enhanced CT images and has superior performance compared with other CNN methods.

## 1. Introduction

Thyroid nodules are one or more agglomerates with abnormal organizational structure in the thyroid gland due to various causes. These nodules are the most common nodular lesions in the human population, with a total incidence of 19% to 46% [1]. The incidence of thyroid cancer has increased 2.4-fold over the past 30 years [2]. Ultrasound technology is the most widely employed imaging method for the diagnosis and follow-up of thyroid disorders such as nodules, tumors, and cysts [3, 4]. However, with the further improvement of CT examination and the proposal of multirow spiral CT, contrast-enhanced CT scanning is gradually gaining importance in the diagnosis of thyroid nodules [5–7].

To reduce the influence of subjective factors in physician diagnosis and improve accuracy of diagnosis, many researchers have recently done a lot of research in the direction of computer-aided diagnosis. Traditional computer-aided diagnosis methods usually process various medical images such as ultrasound images, CT images, MRI images, X-ray films, and pathological slice staining images and design algorithms for disease classification or target segmentation. Singh and Jindal [8] first extracted 13 gray-level cooccurrence matrix (GLCM) features and then utilized a support vector machine (SVM) to classify thyroid nodules with a maximum classification accuracy of 84.62%. Nugroho et al. [9] classified thyroid nodules by analyzing the edge features of nodules in ultrasound images with a final accuracy of 92.30%. Iakovidis et al. [10] used local binary patterns (LBPs), fuzzy local binary patterns (FLBPs), and fuzzy gray-level histograms (FGLHs) to train SVMs with polynomial kernels to detect thyroid nodules. The area under the receiver operating characteristic curve (AUC) estimates that the best performance was 97.5%. Although the above studies have achieved good results on ultrasound images, they are not suitable for CT images because of the high complexity of thyroid CT images, small thyroid regions, and artifacts. As shown in the study by Peng et al. [11], the accuracy of the recognition method based on CT image statistical texture features was only 88%. Liu et al. [12] used 17 texture features to train SVM. The accuracy, AUC, sensitivity, specificity, positive predictive value, and negative predictive value were 0.8673, 0.9105, 0.9130, 0.8269, 0.8235, and 0.9146, respectively. In addition, the above methods require manual participation in complex preprocessing steps and feature extraction, all of which make it difficult to further improve the algorithms. How to extract features effectively and choose the right features among all features are difficult issues.

To solve the above difficulties, we consider deep convolutional neural networks (CNNs) in this study. CNN is a classic deep learning model [13–15], usually composed of standard convolutional layers, finally terminated by a fully connected layer. By learning a large amount of image data, CNN can automatically train a suitable convolution filter to extract features from the data. As the number of layers is deepened, CNN can obtain more advanced semantic features from the image data. Therefore, CNN can be applied to various image classification tasks [15–17] and achieve superior performance.

CNN can also be applied to image segmentation tasks. FCN [18] discards the final classification layer of VggNet-16 [19], converts the fully connected layer into a convolutional layer, and acquires feature maps of the last few layers to improve the segmentation accuracy. SegNet [20] replaces the original fully connected layer by constructing a decoder that is symmetric with the encoder and combines the encoder's pooled layer information to obtain the segmentation result of the target. U-Net [21] combines the encoder's feature map information to complement the segmentation details. Recently, CNNs have been explored in detection and segmentation tasks in the medical domain, such as classification of diseases on thyroid SPECT images by CNN [22], lung nodule classification by means of multiscale CNNs [23], mitosis detection in breast cancer histology images by means of a supervised CNN [24, 25], and small organs (e.g., the pancreas) segmentation in abdominal CT scans [26, 27].

However, deep convolutional neural network requires a myriad number of training data to prevent overfitting, which are not usually readily available for most routine medical imaging applications. When it is difficult to obtain enough training data, disassembling a task into a few small tasks based on prior knowledge will help to achieve better results. Xie et al. [28] transferred the image representation abilities of three ResNet50 models to characterize the overall appearance, heterogeneity of voxel values, and heterogeneity of shape of lung nodules, respectively, and jointly utilized them to classify lung nodules, and they achieved a lung nodule classification accuracy of 93.40%, which is markedly higher than the accuracy of the single network method. Zhang et al. [29] proposed a combined deep and handcrafted visual feature (CDHVF) based algorithm that uses features learned by three finetuned and pretrained deep convolutional neural networks (DCNNs) and two handcrafted descriptors in a joint approach. The CDHVF algorithm achieved an accuracy of 85.47% on the ImageCLEF 2016 Subfigure Classification dataset, which is higher than the best performance of other purely visual or deep approaches.

**Figure 1:**
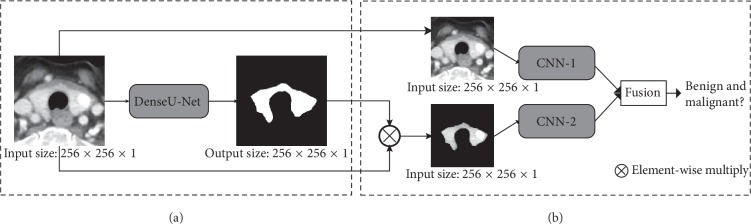
Flowchart of the proposed method for detection of thyroid nodules.

The method for detecting benign and malignant thyroid nodules proposed in this study consists of two parts, as shown in Figure 1. In the first part, the region of interest in the thyroid CT image is automatically segmented by a modified U-Net (DenseU-Net). The second part is the fusion of two different CNN network structures (CNN-F). CNN-1 uses the original CT image for training, and CNN-2 uses the CT image processed by segmentation mask for training. Finally, we merge the two networks into CNN-F and combine various feature levels to identify benign and malignant thyroid nodules.

The main contributions of this study are the following:

1. An innovative network named DenseU-Net, which is based on improved U-Net, is proposed for segmentation of thyroid contrast-enhanced CT images
2. A new method is developed to detect benign and malignant thyroid nodules, which applies transfer learning and fuses multiple levels of features by fusing two different CNN structures
3. No complex preprocessing of thyroid contrast-enhanced CT images is required in the study, and objective and encouraging performance is achieved without any user intervention

## 2. Materials and Methods

### 2.1. Datasets and Materials

The thyroid contrast-enhanced CT images used in this study were provided by a local hospital. Nonionic contrast media were used for radiography; the concentration was 300 mgl/ml, the flow rate was 3 ml/s, the dose was 1 ml/kg, the forearm was injected intravenously, the injection rate was 2.5∼3 ml/s, and the arterial phase was scanned with a delay of 25∼30 s. There are 2012 CT images from 398 patients: 73 patients were diagnosed with malignant thyroid nodules, for a total of 591 CT images, and 325 patients had benign thyroid nodules, for a total of 1421 images. The final diagnosis of these images was based on fine-needle aspiration (FNA) biopsy, and unless the patient underwent surgery, the FNA results were used as a basis for the facts, so the data can be considered accurate. In addition, the above CT images were adjusted by a professional physician to the proper window width and window level so that the thyroid nodule can be clearly observed. The Digital Imaging and Communications in Medicine (DICOM) data were normalized to a grayscale image with a grayscale value of 0–255 according to the appropriate window width and window level. The grayscale image format is jpg. The outline of the thyroid region in the CT image was manually drawn by a doctor. A total of 500 CT images were drawn, and each CT image corresponds to a mask.

### 2.2. DenseU-Net Architecture

#### 2.2.1. U-Net as the Basic Architecture

**Figure 2:**
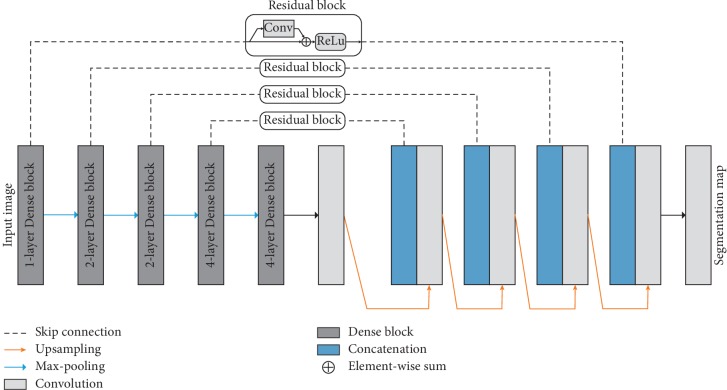
Overall architecture of DenseU-Net.

The DenseU-Net (shown in Figure 2) designed in this study uses the standard U-Net as the basic framework of the network, including the encoder and decoder. The encoder portion is composed of a multilayer convolution layer in which the transferred feature map size is reduced layer by layer, and the number of channels is increased layer by layer. The encoder is represented by the following formula:

(1) $$\begin{array}{c} x_{\ell }=f_{\ell }\left(x_{\ell -1}\right), \end{array}$$

where *f*_*ℓ*_ represents a series of convolution operations for each layer. **x**_*ℓ*_ represents the output of layer *ℓ*. The specific *f*_*ℓ*_ formula is as follows:

(2) $$\begin{array}{c} X_{\ell }=\text{ReLU}\left(W_{\ell }\;⊗\;X_{\ell -1}+B_{\ell }\right), \end{array}$$

where *W*_*ℓ*_ is the convolution kernel weight, *B*_*ℓ*_ is the offset value, and ⊗ is the convolution operation. And ReLU is the linear rectification functions, the formula is as follows:

(3) $$\begin{array}{c} f\left(x\right)=\max\left(0,x\right). \end{array}$$

Each layer of the decoder portion is composed of an upsampling layer and a convolutional layer, wherein the transmitted feature map size is increased layer by layer, and the number of channels is reduced layer by layer. The feature map generated by each layer in the encoder is connected with the corresponding layer in the decoder. The decoder obtains the edge feature of the segmented object, layer by layer, in a rough to fine manner by acquiring decoder feature maps of different scales. Thus, different layering features of the encoder can be incorporated into the decoder, making the network more accurate and scalable. The decoder part is expressed in the following formula:

(4) $$\begin{array}{c} x_{l+n}=f_{l+n}\left(\left[x_{l-n},x_{l+n}\right]\right). \end{array}$$

#### 2.2.2. Dense Learning Mechanism

**Figure 3:**
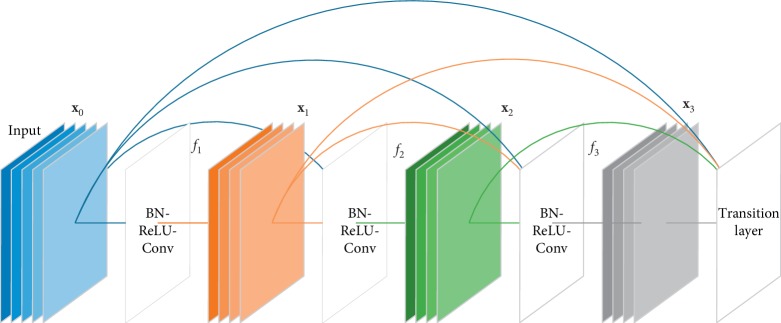
Architecture of Dense block.

In deep convolutional neural networks, the depth of the network is a very important parameter. In general, as the network deepens, the learning performance of the network becomes better and better. However, what follows is the problem of gradient disappearance, resulting in worse network training results. To overcome this problem, Huang et al. [30] proposed the dense convolutional network (DenseNet). Inspired by DenseNet, this study replaces the convolutional network layer of the encoder part with the Dense block. The Dense block is represented in the following formula:

(5) $$\begin{array}{c} x_{\ell }=f_{\ell }\left(\left[x_{0},x_{1},\ldots ,x_{\ell -1}\right]\right), \end{array}$$

where *f*_*ℓ*_ represents a series of convolution operations at layer *ℓ*. [**x**_0_, **x**_1_,…, **x**_*ℓ*−1_] indicates the parallel connection of the output of the first *ℓ* − 1 layer in the Dense block. In a Dense block, the input for each layer comes from the output of all the layers above (shown in Figure 3). Because of this design, the DenseU-Net encoder is narrower and has fewer parameters. The number of output feature maps per convolution layer in the dense block is very small, and this connection method makes the transmission of features and gradients more efficient, and the DenseU-Net is easier to train. At the same time, because the parameter quantity is much less than the general U-Net, it has a certain inhibitory effect on overfitting and has a certain regularization effect.

The DenseU-Net encoder part modified by the Dense block is expressed in the following formula:

(6) $$\begin{array}{c} x_{\ell }=g_{\ell }\left(\left[x_{0},x_{1},\ldots ,x_{\ell -1}\right]\right). \end{array}$$

Specifically, the algorithm *g*_*ℓ*_ is a design of BN-ReLU-Conv ((1 × 1) × (4 *∗* growth rate))-BN-ReLU-Conv ((3 × 3) × (growth rate)). In this paper, three different Dense blocks are used according to the U-Net network structure, and the growth rates are 32, 64, and 128 respectively. This is designed to improve the overall performance of the network when the depth of the encoder is too deep. When the growth rate is set to 32, the encoder block on the fifth layer of the encoder will have 16 layers, which is much larger than the number of layers in the decoder part, and the encoder and decoder layers are seriously unbalanced, affecting the overall performance of the network.

#### 2.2.3. Residual Block as Skip Connection

The context information is directly connected to the decoder in standard U-Net although this connection preserves the most primitive context information to help the decoder reconstruct the contour and detail of the segmentation target. On the other hand, these unprocessed feature maps also contain a large number of interference features, such as segmentation information outside the target area, which will reduce the performance of the network. To solve this problem, we used the Residual block in ResNet [31] to replace the original connection. The Residual block is represented by the following formula:

(7) $$\begin{array}{c} x_{\ell }=f_{\ell }\left(x_{\ell -1}\right)+x_{\ell -1}, \end{array}$$

where *f*_*ℓ*_ represents the convolution operation in the Residual block and **x**_*ℓ*−1_ represents the output of the previous layer. In the Residual block, an input branch is processed by *f*_*ℓ*_ and added to the original input. This preserves the necessary information of the original feature map and can filter out the useless interference features under the processing of *f*_*ℓ*_. The DenseU-net decoder portion modified by the Residual block is expressed in the following formula:

(8) $$\begin{array}{c} x_{\ell +n}=f_{\ell +n}\left(\left[f_{\ell -n}\left(x_{\ell -n}\right)+x_{\ell -n},x_{\ell +n}\right]\right). \end{array}$$

### 2.3. CNN-F Architecture

**Figure 4:**
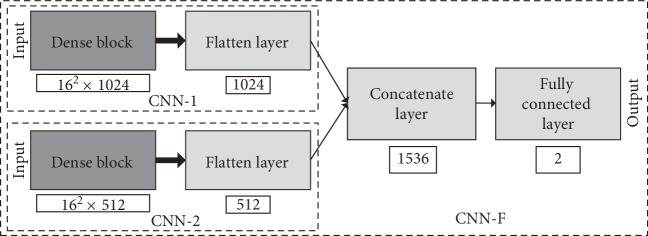
Architecture of CNN-F. The CNN-1 and CNN-2 classification layers in CNN-F were abandoned.

By learning a large amount of image data, CNN can train a suitable convolution filter to extract various features of the image. Therefore, for different types of images, training the corresponding convolution filter can extract the features of the image more effectively. In addition, different CNN architectures can learn different characteristics: shallow networks are suitable for learning low-level features and deep networks can learn advanced features after being fully trained. Moreover, as the number of network layers increases, CNN can learn more complex functions. Therefore, in this paper, CNN-F (shown in Figure 4) is proposed for the fusion of shallow and deep networks. CNN-F consists of CNN-1 and CNN-2 in parallel. Both image data are simultaneously input to CNN-1 and CNN-2 from the input. The image features extracted by the two networks are fused by the concatenate layer, and the result is finally output by the fully connected layer whose activation function is softmax. This structure can capture all features of thyroid nodules and the complex high-level features of the surrounding tissue with CNN-1 and use CNN-2 to capture the subtle low-level texture features inside the thyroid gland so that multiple feature levels can be learned from contrast-enhanced thyroid CT images.

**Figure 5:**
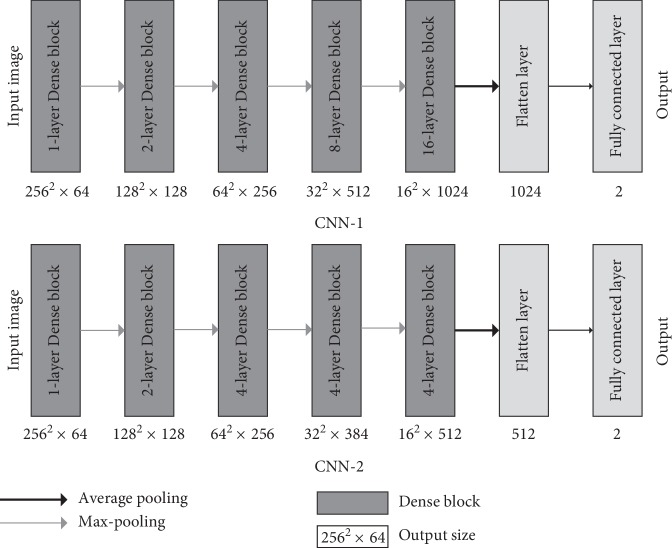
Architecture of CNN-1 and CNN-2.

The detailed structure of the deep network CNN-1 in Figure 4 is shown in Figure 5. This network uses the DenseNet structure to extract features from the input image and finally classifies it by a layer of fully connected layers and softmax functions.

Specifically, CNN-1 has five Dense blocks with a growth rate of 32 and a total of 63 convolutional layers. The activation functions used by all convolutional layers are linear rectification functions (ReLU). In addition, other types of layers are used in CNN-1. After each Dense block, the maximum pooling layer with a window size of 2 × 2 and a stride of 2 is processed and the size of the feature map is reduced by half after processing. Also, CNN-1 applies batch normalization [32] after all convolutional layers, which renormalizes the activation value of the previous layer on each batch so that the mean of its output data is close to zero and the standard deviation is close to 1. Batch normalization works to accelerate convergence, control overfitting, reduce the sensitivity of the network to initialization weights, and allow for larger learning rates. There is a layer of average pooling behind all convolutional layers to reduce the feature size of each channel to 1, followed by the Flatten layer, which “flattens” the input feature map to one dimension for transition from the convolutional layer to the fully connected layer. At the end of CNN-1 is a layer of fully connected layers with two nodes, using the softmax function to generate predicted labels.

The overall structure of CNN-2 and CNN-1 is the same, and the two are different in the total number of layers. CNN-2 has 31 convolutional layers. The 3rd to 5th Dense blocks in CNN-2 are two-layer structures. Its growth rate is 32.

### 2.4. Training Method

Because the training sample has only 500 original images and 500 mask tags, this will easily lead to network overfitting. Therefore, to reduce the impact of less data, real-time data enhancement was used in training to increase data diversity, such as randomness, horizontal flip, random shear stretch, and random rotation. When training DenseU-Net, we first initialized DenseU-Net with random parameters. Second, a 256 × 256 contrast-enhanced CT image of the thyroid was used as the input of DenseU-Net, and the same size mask was used as a label to supervise the network.

The loss function used by DenseU-Net is Dice loss, which is the Loss function proposed by Milletari et al. [33] in V-net. The dice coefficient is derived from the Sørensen–Dice coefficient and is a statistical indicator developed by Thorvald Sørensen and Dice [34] in 1945. It is a set similarity measure function that describes the similarity of two contour regions, equivalent to the F1 score, and its formula is

(9) $$\begin{array}{c} \text{DSC}\left(A,B\right)=2\frac{\left|A\;\cap \;B\right|}{\left|A\right|+\left|B\right|}=\frac{2\text{TP}}{2\text{TP}+\text{FN}+\text{FP}}, \end{array}$$

where *A* and *B* represent the set of points contained in the two contours. Then, Dice loss can be expressed as

(10) $$\begin{array}{c} \text{DL}=1-2\frac{\sum_{n=1}^{N}p_{n}t_{n}+\epsilon }{\sum_{n=1}^{N}\left(p_{n}+t_{n}\right)+\epsilon }, \end{array}$$

where *p*_*n*_ and *t*_*n*_ are the values of each pixel of the prediction mask and the real mask, respectively, and the value is between 0 and 1. *ϵ* is the smoothing coefficient, which reduces the loss value and is used to reduce overfitting of the network.

When training CNN-F, the positive and negative samples in the dataset are not balanced, which will lead to slow convergence and performance degradation during the training process. Therefore, this paper oversamples fewer positive samples to match or approximate the number of negative samples. At the same time, to reduce the overfitting effect caused by the small amount of data, this paper pretrained CNN-1 with a set of 25,000 natural images in the ImageNet dataset. The weight of CNN-1 was initialized using 30 iterations. Then CNN-1 was trained with the original contrast-enhanced CT images. Similarly, these CT images were enhanced in real time before entering the network.

For CNN-2, its weight was initialized with random parameters. CNN-2 was then trained with image data containing only the thyroid ROI processed by the mask. In addition, the above networks were all optimized using the Adam algorithm [35]. The loss function used by CNN-1 and CNN-2 is multiclass cross entropy, and the formula is

(11) $$\begin{array}{c} \text{loss}=-\overset{n}{\underset{i=1}{\sum }}{\hat{y}}_{i1}\log\;y_{i1}+{\hat{y}}_{i2}\log\;y_{i2}+\cdots +{\hat{y}}_{im}\log\;y_{im}, \end{array}$$

where *n* is the number of samples and *m* is the number of classifications.

After training CNN-1 and CNN-2, the two were combined in CNN-F. In detail, we first locked all convolutional layers of CNN-1 and CNN-2 and then trained the weights of the fully connected layers in CNN-F. Finally, the convolutional layers of CNN-1 and CNN-2 were unlocked in a back-to-front manner, and we finetuned the unlocked convolutional layer. The purpose is to slowly adjust the trained weights without destroying the weights.

### 2.5. Performance Measurement

In this study, we performed five-fold cross validation on the thyroid segmentation dataset and the benign and malignant datasets. Four of them were used to train the network, one was used to verify the performance of the evaluation model, and data from the same patient were prevented from being separated into the training set and the validation set on the benign and malignant datasets.

The evaluation method of DenseU-Net is the Dice coefficient, and the value of the Dice coefficient is between 0 and 1. The closer the value is to 1, the more accurate the segmentation is.

To evaluate our proposed CNN-F, we used the classification recall, precision, accuracy, specificity, F1 score, and the area under the receiver operating characteristic (ROC) curve (AUC) [36] to evaluate the performance of different methods.

It can be determined if the classification results of the CNN are correct and if the sample is positive, true positive (TP), true negative (TN), false positive (FP), or false negative (FN) for each class.

Recall, precision, accuracy, and F1 score are defined as follows:

(12) $$\begin{array}{c} \text{accuracy}=\frac{\text{TP}+\text{TN}}{\text{TP}+\text{TN}+\text{FN}+\text{FP}},\\ \text{recall}=\frac{\text{TP}}{\text{TP}+\text{FN}},\\ \text{precision}=\frac{\text{TP}}{\text{TP}+\text{FP}},\\ \text{specificity}=\frac{\text{TN}}{\text{TN}+\text{FP}},\\ \text{F}1\text{ score}=\frac{2\text{TP}}{2\text{TP}+\text{FN}+\text{FP}}. \end{array}$$

Accuracy is the ratio of correctly predicted observations. Recall is the ratio of the number of samples that are correctly predicted for the class to the total number of samples for the class and is also called the sensitivity or hit rate. Precision refers to the ratio of the number of category samples correctly predicted to the total number of samples predicted for that category. Specificity is the ratio of correctly predicted negative samples to the total negative samples. F1 score is the harmonic mean of precision and sensitivity of the classification. The larger these performance values are, the better the performance of a method is.

## 3. Experiments and Results

### 3.1. Implementation Details

During training, the objective function used by DenseU-Net is the Dice loss function. The optimization method is Adam [35], in which the learning rate of Adam is set to 0.0001, the exponential decay rate of first-order moment estimation is set to 0.9, and the exponential decay rate of the second moment estimate is set to 0.999. In addition, the training data are entered into the network in batches of two.

The objective function adopted by CNN-1 is multiclass cross entropy function. In pretraining, the optimization method is Adam [35], in which the learning rate of Adam is set to 0.001 and the exponential decay rate of first-order moment estimation is set to 0.9. The exponential decay rate of the second-order moment estimation is set to 0.999. Then, when finetuning CNN-1, the stochastic gradient descent optimization algorithm (SGD) is adopted. The learning rate of the SGD is set to 0.00001 and the momentum parameter is 0.9. The objective function adopted by CNN-2 is also a multiclass cross entropy function, in which the learning rate of Adam is set to 0.001, the exponential decay rate of first-order moment estimation is set to 0.9, and the exponential decay rate of second-order moment estimation is 0.999. Finally, the CNN-F objective function is also a multiclass cross entropy function. The optimization method is the stochastic gradient descent optimization algorithm (SGD), and the learning rate is set to 0.00001.

Other CNN methods, including VGG-16 [19], ResNet50 [31], and InceptionV3 [37], were also implemented for comparison with the methods proposed in this paper. The training method details are the same as described above.

All experiments were conducted on a personal computer. The computer has an Intel Core i5-7400 (3.0 GHz) CPU processor, 8 GB RAM, 11 GB Nvidia Geforce GTX1080TI graphics processor, and a 64-bit Windows 10 operating system. The runtime environment is Python 3.5 and Keras with TensorFlow as the back end. The time to train DenseU-Net is about 5 hours. It takes about 4.75 hours to train CNN-1 and CNN-2. It takes 0.062 seconds to split a thyroid CT image on the trained DenseU-Net, and it takes about 0.033 seconds to detect an image on the CNN-F.

### 3.2. Performance of DenseU-Net

**Figure 6:**
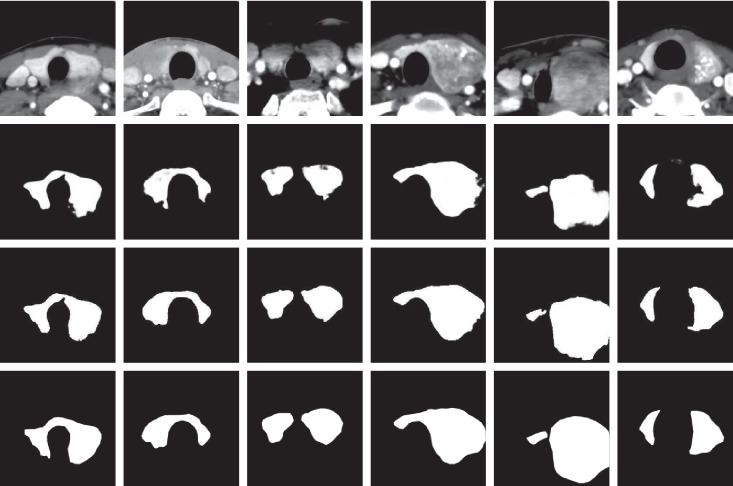
First row is the original enhanced CT image, the second row is the mask generated by U-Net, the third row is the mask generated by DenseU-Net, and the fourth row is the ground truth. The first three columns are benign thyroid nodules images, and the last three columns are malignant thyroid nodules images.

**Figure 7:**
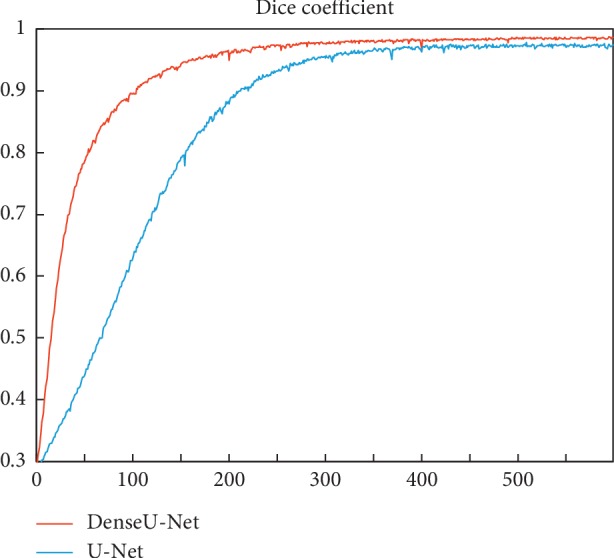
Dice coefficient of U-Net and DenseU-Net on the training set curve with the number of iterations. The U-Net has a Dice coefficient of 0.978 in the training set and a Dice coefficient of 0.945 in the validation set. The Dice coefficient of DenseU-Net is 0.987 in the training set and 0.955 in the validation set.

This paper first studies the performance of the improved DenseU-Net network. The detailed configuration of DenseU-Net is described in Table 1.  Figure 6 shows the segmentation probability plot generated by U-Net and DenseU-Net. At the same time, the research evaluates the performance of DenseU-Net and U-Net by comparing the Dice coefficients. The Dice coefficient curves of the two are shown in Figure 7.

### 3.3. Performance of CNN-F and Comparison with Other Methods

**Figure 8:**
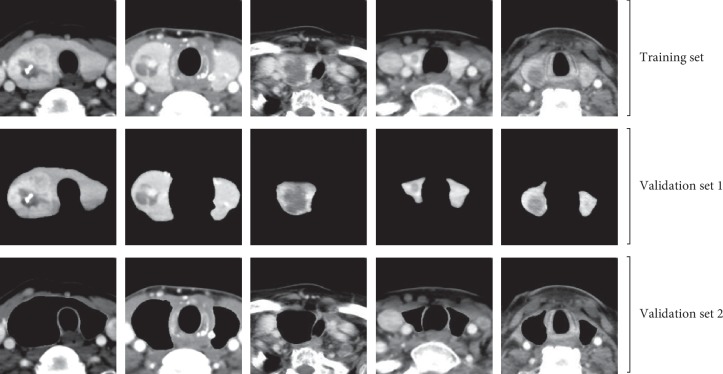
Dataset used in cover-contrast test.

**Figure 9:**
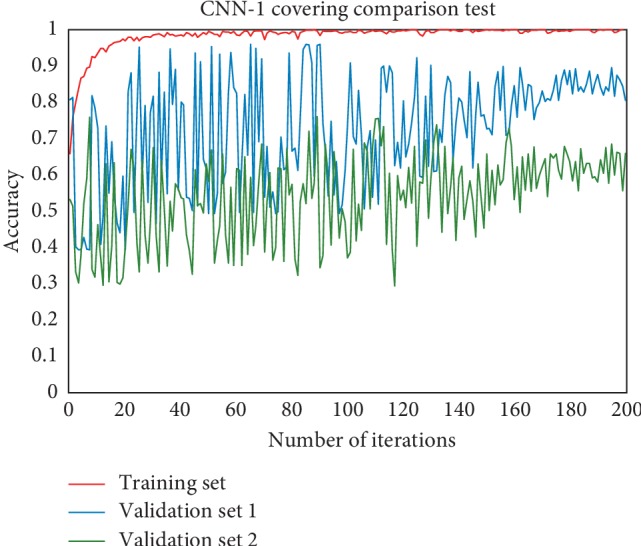
Accuracy of different datasets curve with the number of iterations. In the last twenty rounds, the average accuracy of validation set 1 was 85.10%, and the average accuracy of validation set 2 was 62.38%.

In this study, we used unprocessed raw CT images as training data for CNN-1. To verify that CNN-1 learned the features of the thyroid nodules from the thyroid region, not the rest of the organ tissue in training, we constructed a cover-contrast test. As shown in Figure 8, the training set was composed of original CT images. Validation set 1 was composed of ROI images processed by DenseU-Net, and validation set 2 was composed of CT images from which the thyroid region was removed. The accuracy of the performance with different iteration numbers is shown in Figure 9.

**Figure 10:**
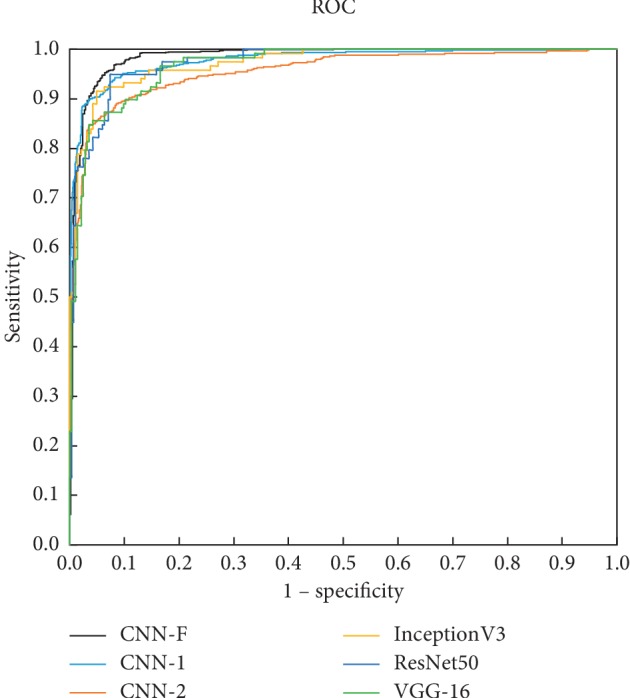
ROC curves for the different models used in this study.

To evaluate our CNN-F, we implemented several other classic CNN methods, including VGG-16 [19], ResNet50 [31], and InceptionV3 [37]. Our CNN-1 and CNN-2 details are described in Table 2, and the details of VGG-16, ResNet101, and InceptionV3 are described in [19, 31, 37]. Respectively, to make it easy, these CNN architectures all performed a 5-fold cross validation. The accuracy, recall, precision, specificity, and F1 score of these architectures with 95% confidence intervals and AUC are reported in Table 3, and *P* < 0.05 is considered to be statistically significant.  Figure 10 shows the ROC curves for different CNNs used to detect thyroid nodules.

## 4. Discussion

Detection of benign and malignant thyroid nodules plays a crucial role in optimal treatment quality and patient outcome. However, due to CT artifacts, complex tissues around the thyroid gland, and blurred edges, traditional machine learning algorithms have difficulty in coping with the detection of thyroid nodules in contrast-enhanced CT. In traditional machine learning, inaccurate image preprocessing will produce potential errors and unreasonable feature selection when manually extracting feature levels leads to classification bias. As shown in Table 3, the first-order texture features, including entropy, uniformity, average intensity, standard deviation, kurtosis, and skewness, were calculated from each ROI after reducing photon noise. Finally, SVM analysis was applied for classification. The accuracy, sensitivity, and specificity were 0.880, 0.821, and 0.933, which were not good enough. However, deep learning method addresses these issues by automatically generating weights and deviations from the data, which generate data-driven, task-specific, and dense feature extractors that take full advantage of the 2D structure in the image. In this study, we solved the problem of thyroid nodule detection based on contrast-enhanced CT through a cascade CNN-based method. At the same time, in order to reduce the overfitting caused by insufficient training data, we trained two networks, respectively, to process the overall appearance of the thyroid gland and the internal texture of the thyroid nodules. To the best of our knowledge, although there have been studies based on deep learning thyroid nodule detection, ours is the first attempt to apply CNN to detection of benign and malignant thyroid nodules in CT images. In this study, DenseU-Net and CNN-F can automatically extract valid features from contrast-enhanced CT images without making any assumptions about the relevant visual features. Specifically, CNN uses a well-trained convolution filter to extract features of different edges, different shapes, and different multilevel textures and combine and normalize them to identify the benign and malignant thyroid gland. At the same time, by learning CT images with artifacts, CNN can also deal with the common bad conditions in CT systems.

To evaluate the proposed DenseU-Net, a 5-fold cross validation was performed in the experiment by using 500 CT images and corresponding segmentation masks. Figure 7 shows that DenseU-Net is significantly better than the original U-Net, both in terms of training speed and final performance. The Dice coefficient has increased from 0.945 to 0.955. Different from those studies [26, 27] that have achieved great results by improving training and optimization methods, this work focused on the optimization of network structure design. The dense connection in DenseU-Net accelerates the training speed and improves the segmentation accuracy combined with the Residual block.  Figure 6 shows the effect of the U-Net and DenseU-Net generating masks on CT images. The results show that the improved network in this paper performs better in thyroid region edge processing than U-Net. Although U-Net completes the task of the thyroid region segmentation well, in some cases, the edge is incorrectly segmented and the mask mistakenly appears inside the thyroid region.

To verify that the features extracted by CNN-1 are valid and are mainly from the thyroid gland, we performed a cover-contrast experiment. As can be seen in Figure 9, CNN-1's weights trained from the original CT image yielded an accuracy of 85.28% on a validation set consisting of thyroid region CT images, while CNN-1 performed poorly on the dataset with the thyroid region removed, with an accuracy rate of about 62.53%, close to random guess. The performance of the ROI validation set was not very good because the segmented thyroid images lost most of the edge information that can provide some useful features for the diagnosis of thyroid nodules. For example, whether the thyroid edge is blurred is an important factor in benign and malignant detection. Also, the validation set 1 loses the other organ region outside the thyroid gland, which can help CNN-1 predict correct results in some respects. The final accuracy of the validation set 2 (62.38%) is still higher than 50%, and the reason may be that the images in the validation set 2 have the thyroid shape information and the other organ region outside the thyroid gland. According to professional doctors, some malignant nodules can cause the thyroid to expand to several times the original size (as shown in Figure 6), and this situation is less when the thyroid nodule is benign. Therefore, the shape information of the thyroid is one of the features learned by CNN-1, making the accuracy of the validation set 2 higher than 50%. It can be seen that the features extracted by CNN-1 after learning the original CT image mainly come from the thyroid region and small part features come from the other organ region outside the thyroid gland and edge of the thyroid. This can also be seen in Table 3. The accuracy of CNN-2 using ROI training data was 93.14%, which is less than the accuracy of CNN-1 using the original training data (94.98%). Although the segmented thyroid CT image loses the edge and the other organ region outside the thyroid gland information, it can make CNN-2 more focused on learning the internal texture features and the appearance of the lesion. Therefore, the fusion of CNN-1 and CNN-2 combines the advantages of both to obtain better detection results.

Finally, to verify that our proposed method can deal well with the benign and malignant detection of thyroid nodules, we compared our method with VGG-16, ResNet50, and InceptionV3. The results in Table 3 and Figure 10 show that our cascade and fusion of multitask convolutional neural networks is superior to other single network in detecting thyroid nodules. In addition, although CNN-2 did not perform as well as other methods, it was excellent in its performance after being fused with CNN-1 because it focused more on the internal texture of the thyroid gland.

**Figure 11:**
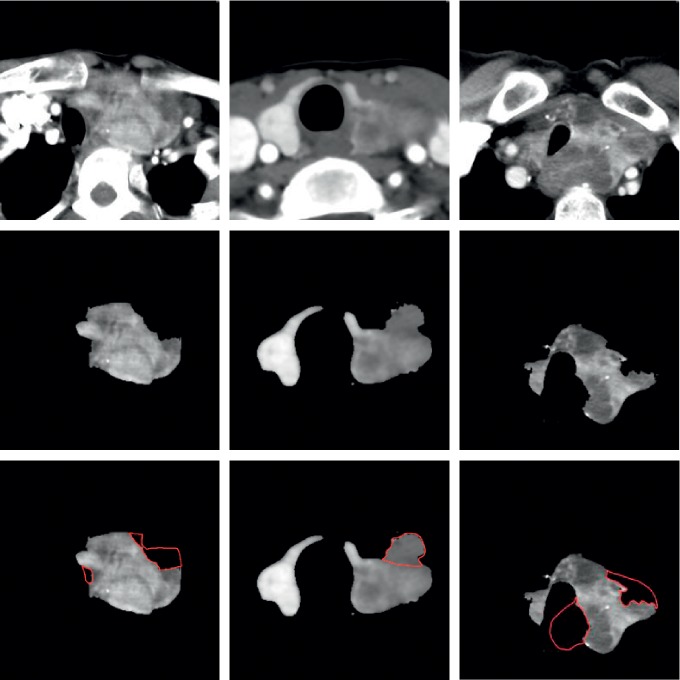
Some ROI examples that are not accurately segmented.

This study did not determine the CNN hyperparameters (such as number of layers and units and filter size) analytically, but mainly empirically. Our proposed DenseU-Net does not effectively segment the thyroid ROI when the background is complex and the lesion is integrated with the surrounding tissue, as shown in Figure 11. Moreover CNN-F cannot accurately judge CT images at the benign and malignant junctions. Therefore, in the future, after obtaining more data, further research on higher performance levels is needed.

## 5. Conclusion

The method of detecting benign and malignant thyroid nodules based on contrast-enhanced CT images plays an important role in assisting doctors in making accurate and correct diagnoses. To solve the problem of benign and malignant identification of thyroid CT images, this paper proposes a method of using the DenseU-Net network for automatic segmentation and then using CNN-F which combines two deep neural network structures and feature selection to perform benign and malignant nodule detection. The DenseU-Net based on U-Net is improved in this paper and is superior to the original network in all aspects. The addition of the Dense block reduces the overall parameter of the network and reduces the overfitting phenomenon to some extent. At the same time, due to the mechanism of Dense Connection, the descending gradient can be effectively transmitted to the deep network layer during the training process so that the convergence speed is better than the original network. In the end, DenseU-Net is able to complete the thyroid region segmentation task. In the task of judging benign and malignant thyroid nodules, this paper proposes a CNN-based detection method, which is composed of two separately trained CNNs, CNN-1 and CNN-2. By letting CNN-1 train on the original CT image and letting CNN-2 learn on the segmented thyroid CT image, CNN-F, which combines the advantages of both, can make better judgments on benign and malignant nodules from multiple feature levels. The results show that the CNN-based method can solve the problem of benign and malignant detection of thyroid nodules and proves its potential clinical application. This research method can provide doctors with an objective second opinion to reduce misdiagnoses caused by excessive fatigue. We recognize that our datasets are not sufficient to learn advanced features and achieve greater accuracy through deep CNN. Therefore, in future research, it is necessary to further test the actual performance level and robustness of the scheme. In addition, we will explore other CNN-based models for more accurate automated detection of thyroid nodules.

## Figures and Tables

**Table 1:** Architecture of DenseU-Net, where Dense-1_/32_ represents a Dense block structure, and its growth rate is 32.

Encoder	Output size	Skip connection	Decoder	Output size
Input	256^∧^2 × 1		Conv6	256^∧^2 × 1
Dense-1_/32_	256^∧^2 × 64	Res1	Conv5	256^∧^2 × 2
Pooling	128^∧^2 × 64		Up4	256^∧^2 × 64
Dense-2_/32_	128^∧^2 × 128	Res2	Conv4	128^∧^2 × 64
Pooling	64^∧^2 × 128		Up3	128^∧^2 × 128
Dense-2_/64_	64^∧^2 × 256	Res3	Conv3	64^∧^2 × 128
Pooling	32^∧^2 × 256		Up2	64^∧^2 × 256
Dense-4_/64_	32^∧^2 × 512	Res4	Conv2	32^∧^2 × 256
Pooling	16^∧^2 × 512		Up1	32^∧^2 × 512
Dense-4_/128_	16^∧^2 × 1024		Conv1	16^∧^2 × 512

Pooling indicates that the maximum pooling layer has a pooling window of 2^∧^2 and a step size of 2. Conv represents the convolutional layer. Up indicates the upsampling layer. Res stands for Residual block.

**Table 2:** Architecture of CNN-1 and CNN-2 in this study.

Layer	Input size	CNN-1	Input size	CNN-2
Conv_1	256 × 256 × 1	3 × 3, 32	256 × 256 × 1	3 × 3, 32
Dense-block_1	256 × 256 × 32	$1\times \left\{\begin{array}{c} 1\times 1,128\\ 3\times 3,32\\ \text{concatenate} \end{array}\right.$	256 × 256 × 32	$1\times \left\{\begin{array}{c} 1\times 1,128\\ 3\times 3,32\\ \text{concatenate} \end{array}\right.$
Max-pooling_1	256 × 256 × 64	2 × 2, stride 2	256 × 256 × 64	2 × 2, stride 2
Dense-block_2	128 × 128 × 64	$2\times \left\{\begin{array}{c} 1\times 1,128\\ 3\times 3,32\\ \text{concatenate} \end{array}\right.$	128 × 128 × 64	$2\times \left\{\begin{array}{c} 1\times 1,128\\ 3\times 3,32\\ \text{concatenate} \end{array}\right.$
Max-pooling_2	128 × 128 × 128	2 × 2, stride 2	128 × 128 × 128	2 × 2, stride 2
Dense-block_3	64 × 64 × 128	$4\times \left\{\begin{array}{c} 1\times 1,128\\ 3\times 3,32\\ \text{concatenate} \end{array}\right.$	64 × 64 × 128	$4\times \left\{\begin{array}{c} 1\times 1,128\\ 3\times 3,32\\ \text{concatenate} \end{array}\right.$
Max-pooling_3	64 × 64 × 256	2 × 2, stride 2	64 × 64 × 256	2 × 2, stride 2
Dense-block_4	32 × 32 × 256	$8\times \left\{\begin{array}{c} 1\times 1,128\\ 3\times 3,32\\ \text{concatenate} \end{array}\right.$	32 × 32 × 256	$4\times \left\{\begin{array}{c} 1\times 1,128\\ 3\times 3,32\\ \text{concatenate} \end{array}\right.$
Max-pooling_4	32 × 32 × 512	2 × 2, stride 2	32 × 32 × 384	2 × 2, stride 2
Dense-block_5	16 × 16 × 512	$16\times \left\{\begin{array}{c} 1\times 1,128\\ 3\times 3,32\\ \text{concatenate} \end{array}\right.$	16 × 16 × 384	$4\times \left\{\begin{array}{c} 1\times 1,128\\ 3\times 3,32\\ \text{concatenate} \end{array}\right.$
Average-pooling	16 × 16 × 1024	16 × 16	16 × 16 × 512	16 × 16
Fully connected layer	1024	2	512	2
Output	2		2	

Here, “conv” denotes convolutional layer. Number formats of CNN-1 and CNN-2 are all: convolution kernel size, number of convolution kernels.

**Table 3:** Performance comparison of different methods (percent).

Method	Accuracy	Recall	Precision	Specificity	F1 score	AUC	CI
CNN-F	**95.73**	87.14	**98.10**	**99.30**	**92.30**	**98.49 (0.0029)**	**[97.91 99.06]**
CNN-1	94.98	**88.66**	93.91	97.61	91.21	97.83 (0.0033)	[97.18 98.49]
CNN-2	93.14	84.10	91.87	96.90	87.81	95.63 (0.0052)	[94.60 96.66]
ResNet50	93.28	82.20	94.17	97.89	87.78	97.41 (0.003)	[97.26 98.28]
InceptionV3	93.78	87.29	91.15	96.48	89.18	97.31 (0.0054)	[96.85 98.46]
VGG-16	92.79	83.90	90.83	96.48	87.22	96.82 (0.0046)	[96.33 97.31]
Peng et al. [11]	88.80	82.10	91.70	93.30	—	95.30	—
Liu et al. [12]	86.73	91.30	82.35	82.96	—	91.05	—

Number format of AUC: mean (standard deviation).

## Data Availability

The data used to support the findings of this study are unavailable from the corresponding author upon request.
